# Role of Novel Inflammatory Factors in Central Retinal Vein Occlusion with Macular Edema

**DOI:** 10.3390/medicina60010004

**Published:** 2023-12-19

**Authors:** Kanako Yasuda, Hidetaka Noma, Tatsuya Mimura, Ryota Nonaka, Shotaro Sasaki, Akemi Ofusa, Masahiko Shimura

**Affiliations:** 1 Department of Ophthalmology, Hachioji Medical Center, Tokyo Medical University, 1163, Tatemachi, Hachioji 193-0998, Japan; kana6723@yahoo.co.jp (K.Y.); nort310317@gmail.com (R.N.); hanshinchelsea@yahoo.co.jp (S.S.); h-ort@tokyo-med.ac.jp (A.O.); masahiko@v101.vaio.ne.jp (M.S.); 2 Department of Ophthalmology, Teikyo University School of Medicine, 2-11-1 Kaga, Itabashi-ku, Tokyo 173-8606, Japan; mimurat@med.teikyo-u.ac.jp

**Keywords:** anti-VEGF therapy, central retinal vein occlusion, CXCL-16, endocan-1, FMS-related tyrosine kinase 3 ligand, macular edema

## Abstract

*Background and Objectives*: To investigate associations among the aqueous humor levels of novel inflammatory factors, including FMS-related tyrosine kinase 3 ligand (Flt-3L), fractalkine, CXC chemokine ligand 16 (CXCL-16), and endocan-1; the severity of macular edema in central retinal vein occlusion (CRVO); and the prognosis of CRVO with macular edema after antivascular endothelial growth factor (VEGF) therapy. *Materials and Methods*: Aqueous humor was obtained during anti-VEGF treatment with intravitreal ranibizumab injection (IRI) in patients with CRVO and macular edema (*n* = 19) and during cataract surgery in patients with cataracts (controls, *n* = 20), and the levels of VEGF and novel inflammatory factors were measured. Macular edema was evaluated by central macular thickness (CMT) and neurosensory retinal thickness (TNeuro), and improvement was evaluated by calculating the percentage change in CMT and TNeuro from before to 1 month after IRI. *Results*: The levels of VEGF and the novel inflammatory factors were significantly higher in the CRVO group, and the levels of Flt-3L, CXCL-16, and endocan-1 were significantly correlated with each other and with the aqueous flare value. Baseline levels of Flt-3L, CXCL-16, and endocan-1 had a significantly negative correlation with the change in CMT, and the baseline level of CXCL-16 was significantly negatively correlated with the change in TNeuro. *Conclusions*: Relations among novel inflammatory factors should be further investigated. These findings may help improve understanding of macular edema in CRVO patients and aid the development of new treatments targeting novel inflammatory factors.

## 1. Introduction

Central retinal vein occlusion (CRVO) is related to atherosclerosis and involves ischemia and inflammation [1]. A common symptom of CRVO is macular edema, which is caused by cytokines including vascular endothelial growth factor (VEGF) [1,2,3,4,5]. These cytokines are related to angiogenesis and inflammation, which cause the development and progression of macular edema in CRVO.

Intravitreal treatment with anti-VEGF agents is the first treatment of choice for macular edema associated with CRVO [6,7,8]. However, treatment may not be successful, and macular edema may also re-occur [9,10], suggesting that other factors besides VEGF contribute to macular edema. In a previous study, we identified a range of inflammatory factors that are related to macular edema severity [11], leading us to suggest that inflammation may be involved.

We identified the four novel inflammatory factors as possibly involved in macular edema: FMS-related tyrosine kinase 3 ligand (Flt-3L), fractalkine, CXC chemokine ligand 16 (CXCL-16), and endocan-1. Flt-3L, a transmembrane protein, can also take the form of a soluble protein [12,13]. As a cytokine, it is involved in the differentiation of hematopoietic stem cells and their mobilization. Flt-3L also regulates hematopoiesis and activates dendrites [12,13]. In addition, it causes lymphocyte proliferation [14]. Flt-3L expression is increased in autoimmune and chronic inflammatory responses in various organs [15,16].

Fractalkine, also referred to as CX3CL1, is constitutively expressed in retinal neurons, retinal pigment epithelium, and microvascular endothelial cells in the eye. When released by tissue lesions, fractalkine participates in the recruitment of monocytes and in local inflammation.

CXCL16 plays a role in angiogenesis and in the chemotaxis of endothelial progenitor cells [17]. Furthermore, it may be related to the prognosis of various cancers, bacterial infections, and systemic sclerosis [18,19,20]. 

Endocan-1 (also known as endothelial cell-specific molecule-1) is a soluble proteoglycan [21]. It is expressed in budding vascular endothelium [22,23] and is implicated in angiogenesis [23,24]. 

Currently, no biomarker is available for predicting disease activity in macular edema associated with CRVO. As mentioned above, VEGF is involved, and CRVO is associated with inflammation, so the measurement of VEGF and inflammatory cytokines is useful. Thus, with the aim of investigating the associations among the aqueous humor levels of the novel inflammatory factors Flt-3L, fractalkine, CXCL-16, and endocan-1; the severity of macular edema in CRVO; and the prognosis of CRVO with macular edema after anti-VEGF therapy, the present study measured the aqueous humor levels of VEGF and the novel inflammatory factors in CRVO patients and macular edema to investigate the associations of each inflammatory factor, the severity of macular edema in CRVO patients, and the prognosis of macular edema in CRVO after anti-VEGF therapy.

## 2. Materials and Methods

### 2.1. Patients

**Figure 1:**
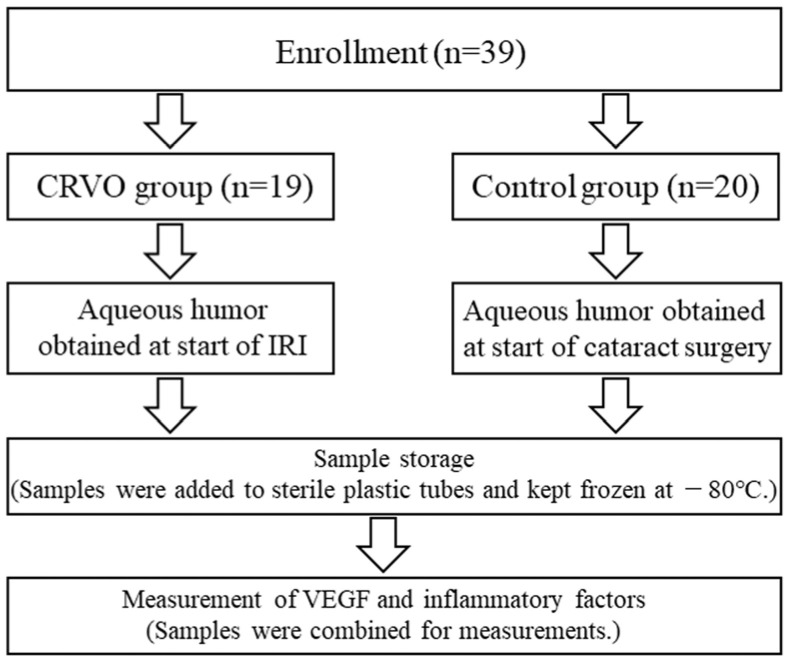
Diagram showing experimental design. CRVO, central retinal vein occlusion; IRI, intravitreal ranibizumab injection; VEGF, vascular endothelial growth factor.

In the Department of Ophthalmology, Tokyo Medical University Hachioji Medical Center (located in Tokyo, Japan), we recruited 19 consecutive CRVO patients and macular edema confirmed by detailed eye examinations and 20 consecutive control patients with cataracts (Figure 1). Patients with cataracts were chosen as the control group because (1) such patients do not have retinal disease, making them suitable as a control for CRVO patients, and (2) aqueous humor can be collected relatively noninvasively during cataract surgery. CRVO patients were treatment-naïve and undergoing initial intravitreal ranibizumab injection (IRI) (Lucentis; 0.5 mg in 0.05 mL; Genentech, Inc., South San Francisco, CA, USA) for macular edema.

The inclusion criteria for CRVO patients were a diagnosis of acute CRVO; logarithm of the minimum angle of resolution (LogMAR) best-corrected visual acuity (BCVA) below 25/30; central macular thickness (CMT) above 300 μm, as measured by spectral-domain optical coherence tomography (SD-OCT) (Spectralis, Heidelberg Engineering, Heidelberg, Germany); the ability to stare at a fixation point for the required time during SD-OCT; and the involvement of the fovea. Exclusion criteria included ischemic CRVO (i.e., capillary non-perfusion in at least 10 areas [25] confirmed by fluorescein angiography [FA]), macular ischemia, type 2 diabetes, diabetic retinopathy, severe cataracts, and other choroidal and retinal diseases. Patients were also excluded if they had received previous ocular treatments, e.g., surgery.

Among other ophthalmologic data, BCVA, SD-OCT findings (refer to below), and aqueous flare values (for more detail, refer to [1]) were taken from medical records.

The ethics committee of Tokyo Medical University approved the study, which complied with the ethical principles of the Declaration of Helsinki. All study participants provided their informed consent in writing.

### 2.2. Collection of Aqueous Humor Samples

First, topical anesthesia was administered. Then, an aqueous humor sample was obtained (mean volume, 0.1 mL) by inserting a 30 G needle into limbus. Samples were added to sterile plastic tubes and kept frozen at −80 °C (Figure 1). 

IRI was administered to the superior temporal quadrant, 3.5 mm from the limbus, with a 30 G needle inserted through the pars plana. After the procedure, patients were instructed to apply antibiotic drops to the treated eye for 3 days. A follow-up evaluation of BCVA, SD-OCT findings (refer to below), and aqueous flare values was performed 4 weeks after IRI. 

In the control group, aqueous humor was obtained by limbal paracentesis during a planned cataract operation and stored in the same way as the samples from the IRI group (Figure 1).

### 2.3. Assessment of Macular Edema

Macular edema was assessed by SD-OCT performed in the week before IRI. Severity was based on CMT (i.e., the distance between the retinal pigment epithelium inner limiting and basal membranes), TNeuro (i.e., subfoveal neurosensory retinal thickness), and SRT (i.e., subfoveal serous retinal thickness) [26,27]. 

### 2.4. Fundus Examination

SD-OCT was also used to examine the fundus by obtaining 6 mm cross-sectional scans (horizontal and vertical). An experienced technician conducted all the scans. First, the technician located the fovea on the images of the fundus. Then, they performed scans centered on the fovea and repeated the scans until highly reproducible images were obtained.

### 2.5. Assessment of Effects of IRI

To assess the effects of IRI on vision, BCVA was measured 1 month after the procedure. In addition, the effects on macular edema were assessed by calculating the change (%ΔX) in CMT and TNeuro as %ΔX = (X_pre_ − X_post_)/X_pre_ × 100 = (1 − X_post_/X_pre_) × 100, where X_pre_ is the pre-treatment and X_post_ is the post-treatment value of CMT or TNeuro.

### 2.6. Assessment of VEGF and Novel Inflammatory Factors

Levels of VEGF and the novel inflammatory factors Flt-3L, fractalkine, CXCL-16, and endocan-1 in aqueous humor were assessed with suspension array (xMAP; Luminex Corp., Austin, TX, USA) [28,29,30,31,32,33] with the Milliplex kit (EMD Millipore, Burlington, MA, USA). The Human Cytokine/Chemokine kit (HCYTO-60K) was used for VEGF, Flt-3L, and fractalkine, and the human Cardiovascular Disease kit (HCVD1MAG-67K) was used for CXCL-16 and endocan-1. Fluorescence-labeled beads were mixed with standards and aqueous humor samples and incubated at 4 °C overnight, as specified by the manufacturer. The next day, detection antibodies were added at room temperature for 1 h, followed by streptavidin–phycoerythrin at room temperature for 30 min. A Luminex 100/200 System determined the readings, and MILLIPLEX Analyst software (5.1 Flex) analyzed the median fluorescent intensity. Concentrations were calculated by a 5-parameter logistic approach. All factors were present at the detectable levels (minimum detectable concentrations: VEGF, 26.3 pg/mL; Flt-3L, 5.4 pg/mL; fractalkine, 22.7 pg/mL; CXCL-16, 13.2 pg/mL; and endocan-1, 11.5 pg/mL).

### 2.7. Statistical Analysis

Data were analyzed with SAS System 9.4 (SAS Institute Inc., Cary, NC, USA) and are shown as means ± SD. We used Student’s unpaired *t* test for normally distributed variables, the Mann–Whitney U test for skewed distribution variables, and Spearman’s rank-order or Pearson’s correlation for intervariable relationships. When the two variables were both continuous and assumed to be normally distributed, we used Pearson’s correlation, and otherwise Spearman’s rank test. The threshold for significance was set at 0.05. Correlation coefficients with an r value of 0.7 and 0.9 were considered to indicate a high correlation, and those with an r value of 0.5 and 0.7 were considered to indicate variables of a moderate correlation.

## 3. Results

### 3.1. Characteristics of the Patients

The mean ± SD age was 63.5 ± 14.9 in the CRVO group (10 men and 9 women) and 68.2 ± 5.1 years in the control group (9 men and 11 women). Neither age (*p* = 0.194) nor sex (*p* = 0.633) were significantly different between the two groups. The mean pre-treatment duration of symptoms was 52.7 ± 48.9 days (range, 5–158 days). In total, 14 of the 19 CRVO patients (73.7%) and 2 of the 20 control patients (10.0%) had hypertension (*p* < 0.01), and 11 of the 19 CRVO patients (57.9%) and 3 of the 20 control patients (15.0%) had hyperlipidemia (*p* = 0.005)

### 3.2. BCVA, SD-OCT Findings, and Aqueous Flare Values after IRI

One month after IRI, we found a significant improvement in BCVA (pre-treatment value, 0.49 ± 0.37 logMAR; post-treatment value, 0.20 ± 0.29 logMAR; *p* < 0.001). In addition, IRI resulted in a significant decrease in CMT (pre-treatment value, 605 ± 182 μm; post-treatment value, 279 ± 146 μm; *p* < 0.001); TNeuro (pre-treatment value, 726 ± 189 μm; post-treatment value, 299 ± 169 μm; *p* < 0.001); SRT (pre-treatment value, 122 ± 112 μm; post-treatment value, 20.2 ± 50.2 μm; *p* < 0.001); and aqueous flare (pre-treatment value, 14.1 ± 7.85 photon counts/ms; post-treatment value, 8.39 ± 5.23 photon counts/ms; *p* = 0.001).

### 3.3. Comparison of Cytokines between CRVO and Control Group

Concentrations of VEGF, Flt-3L, fractalkine, CXCL-16, and endocan-1 (in descending order) were significantly higher in the CRVO group immediately before IRI than in the control group at the start of cataract surgery (Table 1).

### 3.4. Relationship among Cytokines and SD-OCT Findings and Aqueous Flare Values

Baseline relationships between the inflammatory factors, CMT, TNeuro, and SRT, and the aqueous flare value in the CRVO group, are shown in Table 2. CMT was significantly correlated with the aqueous level of VEGF. In this group, significant correlations were found between TNeuro and the level of endocan-1 and between the aqueous flare value and the aqueous humor levels of Flt-3L, CXCL-16, and endocan-1 (Table 2).

### 3.5. Correlations among Each Cytokines in CRVO Group

At baseline, the aqueous Flt-3L level was significantly correlated with the levels of CXCL-16 and endocan-1; the fractalkine level was significantly correlated with the level of CXCL-16; and the CXCL-16 level was significantly correlated with the level of endocan-1 (Table 3).

### 3.6. Correlations between Cytokines and Changes in Clinical Factors

In the CRVO group, the baseline aqueous humor levels of VEGF, Flt-3L, fractalkine, CXCL-16, and endocan-1 showed no correlation with the change in BCVA, but those of Flt-3L, CXCL-16, and endocan-1 showed a significant negative correlation with CMT change (Table 4). The VEGF and fractalkine baseline levels showed no significant correlation with the CMT change (Table 4). Baseline CXCL-16 showed a significant negative correlation with TNeuro change (Table 4), but the changes in VEGF, Flt-3L, fractalkine, and endocan-1 were not significant (Table 4).

## 4. Discussion

This study found that the aqueous levels of VEGF and the novel inflammatory factors Flt-3L, fractalkine, CXCL-16, and endocan-1 were significantly higher in the CRVO group than in the controls. This finding suggests that not only VEGF but also these four inflammatory factors are involved in the pathogenesis of CRVO.

In CRVO, Flt-3L may be associated with a chronic inflammatory response. In fact, our study found a significant correlation between the aqueous flare value and the aqueous level of Flt-3L.

This study found a significant correlation between the aqueous flare value and the aqueous level of CXCL-16 in CRVO patients. We suspect that CXCL16 was upregulated in part because VEGF was expressed by inflammatory cells that had infiltrated the aqueous humor, and we hypothesize that it plays a role in the inflammatory processes of CRVO. However, the exact role of CXCL16 in CRVO needs to be further explored.

VEGF and inflammatory cytokines, which induce hypoxia, may upregulate the expression of endocan-1 [5,21,34,35]. Our finding of significant correlations between the aqueous endocan-1 level and the levels of Flt-3L and CXCL-16 supports an involvement of VEGF and inflammatory cytokines in endocan-1 expression. Endocan-1 has also been proposed to have a role in inflammation [36,37]. In fact, we found that endocan-1 in humor was significantly correlated with the aqueous flare value. The decreased expression of tight junction molecules in the developing retina and hypoxia lead to an increased expression of endocan-1 [38]. In a study in human umbilical vein endothelial cells (HUVECs), endocan-1 reduced the expression of ZO-1 and occludin, increased vascular permeability, and promoted monocyte migration across the endothelium of HUVECs [37]. Consequently, we suggest that the higher endocan-1 expression in hypoxic retina may be linked to the downregulation of the expression of tight junction molecules and an increase in endothelial permeability, i.e., the greater transportation of molecules from the serum across the endothelium. In fact, we found a significant correlation between endocan-1 and TNeuro, suggesting that besides VEGF, endocan-1 might also be involved in inflammation, leading to the development of macular edema associated with CRVO.

Fractalkine, the only CX3C chemokine, is a leukocyte chemoattractant and adhesion molecule [39,40,41,42]. One study suggested that fractalkine may mediate ocular angiogenesis because it promotes chemotaxis and tube formation in endothelial cells and neovascularization of the cornea [43]. Fractalkine may well promote ischemic CRVO because it is known to cause angiogenesis in other types of ischemic retinopathy. Our finding that fractalkine levels were significantly correlated with CXCL-16, a potent angiogenic factor, supports a potential role of fractalkine in the angiogenesis of CRVO. Further investigations are needed to verify the role of fractalkine in CRVO with macular edema.

In this study, BCVA, CMT, TNeuro, SRF, and aqueous flare improved significantly 1 month after IRI. These results are consistent with previous reports [1,44,45,46]. Interestingly, we also found that higher baseline aqueous humor levels of Flt-3L, CXCL-16, and endocan-1 were associated with a poorer subsequent CMT response to IRI at 1 month, and that a higher baseline aqueous humor level of CXCL-16 was associated with worse TNeuro response to IRI at 1 month, suggesting that patients with higher baseline Flt-3L, CXCL-16, and endocan-1 levels have a poor treatment response to IRI. Furthermore, three of the novel inflammatory factors, Flt-3L, CXCL-16, and endocan-1, were significantly correlated with each other and, as mentioned above, with the aqueous flare value. These findings suggest that these three inflammatory factors form an interactive network that influences treatment response to IRI. In addition, because this study showed that these inflammatory factors were significantly negatively correlated with the change in CMT in the first 1 month after IRI, additional anti-inflammatory treatment early in the course of the disease may be useful. However, further study of these factors and treatment response is warranted.

This study has some limitations. First, the time between sampling and onset of symptoms was heterogeneous. The improvement in vision in response to treatment is largely determined by how long the intraretinal fluid has persisted. In the acute phase, bleeding makes it difficult to determine by FA whether CRVO is of the ischemic type. Thus, the time from onset of symptoms to initial treatment may influence how well FA can determine the type of ischemia. Second, the present study did not have a long follow-up period. It would be useful to assess changes in retinal pathology over time, so future studies should add a longer follow-up with assessments of retinal pathology.

## 5. Conclusions

In summary, aqueous levels of VEGF and the novel inflammatory factors Flt-3L, fractalkine, CXCL-16, and endocan-1 were significantly elevated in the CRVO group compared with the control groups. Furthermore, three of the novel factors, Flt-3L, CXCL-16, and endocan-1, were significantly correlated with each other and with the aqueous flare value. The baseline aqueous humor levels of Flt-3L, CXCL-16, and endocan-1 were significantly negatively correlated with the change in macular edema in CRVO patients. These findings suggest the importance of further investigating the relations among these novel inflammatory factors. Our study may improve the understanding of CRVO-related macular edema, which in turn may result in new therapies that target novel inflammatory factors.

## Figures and Tables

**Table 1:** Aqueous humor factors in the control group at the start of cataract surgery and in the central retinal vein occlusion group immediately before intravitreal ranibizumab injection.

	Control (*n* = 20)	CRVO (*n* = 19)	*p* Value
VEGF (pg/mL)	58.1 ± 23.8	121 ± 130	**<0.05**
Flt-3L (pg/mL)	1.01 ± 0.26	10.9 ± 4.63	**<0.05**
Fractalkine (pg/mL)	19.1 ± 2.08	136 ± 268	**<0.05**
CXCL-16 (pg/mL)	241 ± 138	352 ± 161	**<0.05**
Endocan-1 (pg/mL)	286 ± 95.5	1667 ± 1069	**<0.05**

CRVO, central retinal vein occlusion; CXCL-16, CXC chemokine ligand 16; Flt-3L, FMS-related tyrosine kinase 3 ligand; VEGF, vascular endothelial growth factor; *p* values written in bold indicate a significant difference; variables were compared with Student’s *t* test or Mann–Whitney U test.

**Table 2:** Relationships of aqueous humor factors with spectral-domain optical coherence tomography parameters.

	VEGF	Flt-3L	Fractalkine	CXCL-16	Endocan-1
Variable	*r*	*r*	*r*	*r*	*r*
	*p* value	*p* value	*p* value	*p* value	*p* value
CMT	0.58	0.02	0.01	0.29	0.30
	**<0.05**	0.933	0.961	0.231	0.214
TNeuro	0.42	0.23	−0.08	0.41	0.49
	0.075	0.348	0.738	0.080	**<0.05**
SRT	−0.23	0.42	−0.15	0.22	0.33
	0.328	0.066	0.517	0.351	0.160
Aqueous flare	0.10	0.58	0.12	0.64	0.48
	0.549	**<0.05**	0.461	**<0.05**	**<0.05**

CMT, central retinal thickness; CXCL-16, CXC chemokine ligand 16; Flt-3L, FMS-related tyrosine kinase 3 ligand; SRT, serous retinal thickness; TNeuro, thickness of the neurosensory retina; *r*, correlation coefficient; VEGF, vascular endothelial growth factor; *p* values written in bold indicate a significant difference; variables were compared with Spearman’s rank-order or Pearson’s correlation analysis.

**Table 3:** Correlations between inflammatory factors in aqueous humor.

	VEGF	Flt-3L	Fractalkine	CXCL-16	Endocan-1
Variable	*r*	*r*	*r*	*r*	*r*
	*p* value	*p* value	*p* value	*p* value	*p* value
VEGF		−0.12	0.09	−0.01	0.39
		0.615	0.705	0.973	0.097
Flt-3L			0.17	0.77	0.46
			0.309	**<0.05**	**<0.05**
Fractalkine				0.60	0.10
				**<0.05**	0.696
CXCL-16					0.46
					**<0.05**

CXCL-16, CXC chemokine ligand 16; Flt-3L, FMS-related tyrosine kinase 3 ligand; *r*, correlation coefficient; VEGF, vascular endothelial growth factor; *p* values written in bold indicate a significant difference; variables were compared with Spearman’s rank-order or Pearson’s correlation analysis.

**Table 4:** Correlations between aqueous humor levels of growth factors and inflammatory mediators, and changes in clinical factors.

	Improvement in BCVA	Change in CMT	Change in TNeuro
Variable	*r*, *p* value	*r*, *p* value	
VEGF (pg/mL)	0.21, 0.321	0.19, 0.425	0.06, 0.789
Flt-3L (pg/mL)	0.06, 0.783	−0.67, **<0.05**	−0.42, 0.070
Fractalkine (pg/mL)	0.20, 0.338	0.11, 0.647	0.03, 0.890
CXCL-16 (pg/mL)	−0.01, 0.991	−0.51, **<0.05**	−0.47, **<0.05**
Endocan-1 (pg/mL)	−0.04, 0.847	−0.46, **<0.05**	−0.33, 0.172

BCVA, best-corrected visual acuity; CMT, central macular thickness; CXCL-16, CXC chemokine ligand 16; Flt-3L, FMS-related tyrosine kinase 3 ligand; TNeuro, neurosensory retinal thickness; *r*, correlation coefficient VEGF, vascular endothelial growth factor; *p* values written in bold indicate a significant difference; variables were compared with Spearman’s rank-order or Pearson’s correlation analysis.

## Data Availability

The datasets collected and/or analyzed in the present study are available on request from the corresponding author.
